# Perceptions, Utilization, and Impact of Artificial Intelligence on Medical Science Liaisons: A Global Cross-Sectional Survey of Medical Affairs Professionals

**DOI:** 10.7759/cureus.107157

**Published:** 2026-04-16

**Authors:** Samuel J Dyer, Ellen Shannon, Jeff Kraemer

**Affiliations:** 1 Medicine, Medical Science Liaison Society, Miami, USA; 2 Internal Medicine, Pfizer, Baltimore, USA; 3 Medical Affairs, Medical Science Liaison Society, Miami, USA

**Keywords:** artificial intelligence, digital health, healthcare professionals, key opinion leaders, medical affairs, medical science liaisons, pharmaceutical industry

## Abstract

Introduction

Artificial intelligence (AI) is increasingly being adopted in Medical Affairs and has the potential to transform the role of medical science liaisons (MSLs). This study was designed to assess the current state of AI adoption, perceptions, organizational readiness, and potential impact among MSLs through a global survey.

Methods

A global, cross-sectional survey was conducted, including 367 participants from 48 countries representing diverse roles in Medical Affairs, including medical science liaisons (MSLs), MSL leadership, and executive Medical Affairs leadership, across pharmaceutical, biotechnology, medical device, diagnostic, and other healthcare organizations. The survey was distributed through professional networks, including LinkedIn, Medical Science Liaison Society newsletter subscribers, pharmaceutical industry conferences, and online platforms dedicated to Medical Affairs professionals, using a non-probability convenience sampling approach.

Results

Overall, 27% of the respondents currently utilize AI tools for key opinion leader (KOL) engagements. The primary applications included literature review (22%), data analysis (20%), and presentation preparation (16%). The most commonly reported benefits were improved efficiency (42%) and better preparation for KOL engagements (18%). However, only one-third of organizations had established policies governing AI use by MSLs, indicating a gap in governance and organizational readiness. Knowledge of AI varied: 41% of the respondents reported being somewhat knowledgeable, and 30% reported not being very knowledgeable. Despite this, 74% of the respondents believed that AI would be impactful or highly impactful in Medical Affairs and the MSL function, and 87% considered it important or very important to learn about AI technology to remain competitive.

Conclusion

AI adoption among MSLs remains limited, but there is a clear and growing recognition of its potential impact. These findings highlight both the opportunity and the need for improved governance, education, and strategic implementation as AI continues to shape the evolving role of MSLs within Medical Affairs.

## Introduction

Artificial intelligence (AI) has emerged as a transformative force in healthcare, with expanding applications within the pharmaceutical industry across multiple functions, including Medical Affairs [1-4]. As AI technologies continue to evolve, their potential to reshape the role of medical science liaisons (MSLs) has attracted growing interest and debate within pharmaceutical, biotechnology, medical devices, diagnostics, and other healthcare companies [5-7]. The integration of AI into Medical Affairs activities promises to enhance efficiency, improve decision-making processes, and ultimately lead to improved patient outcomes.

There is a growing body of research examining AI adoption across healthcare, with studies highlighting its potential in areas such as diagnostics, clinical research, and medical information services where automation and large-scale data processing can deliver measurable gains [8-11]. However, most of this work focused on clinical or operational domains rather than Medical Affairs. While there is increasing commentary on the opportunities and risks of AI across various roles and functions within healthcare, to our knowledge, no prior study has specifically investigated AI adoption or perceptions of AI within the MSL profession [12-14].

This gap in understanding the specific opportunities and risks of AI within the MSL profession is particularly significant because the role combines data-driven responsibilities, such as literature analysis and insight generation, with highly relational activities, including scientific exchange and engagement with key opinion leaders (KOLs) and other healthcare professionals (HCPs). In contrast to operational roles such as clinical trial site coordination, regulatory operations, or publication management, the MSL role combines scientific credibility with nuanced KOL engagement, so examining how AI is perceived is vital because its use may impact how effectively MSLs deliver value to the KOLs they support.

Thus, the purpose of the survey was to assess the adoption, perceptions, and anticipated impact of AI across Medical Affairs roles, with a particular focus on MSLs, who represent the largest group of respondents and serve a unique role in scientific exchange and KOL engagement. This study is highly relevant to Medical Affairs because it provides unique insights into the evolving landscape of AI adoption and its implications for MSL activities. The findings of this survey will contribute to the growing body of knowledge on AI in healthcare and specifically inform strategies for its integration into Medical Affairs. These insights can help organizations design more effective implementation plans, tailor training programs, and address barriers to adoption while also highlighting areas where AI can enhance MSL activities, such as data analysis, scientific communication, and KOL engagement.

To our knowledge, this is the first comprehensive global survey dedicated to exploring the adoption of AI, its perceptions, and its influence on the MSL profession.

## Materials and methods

Survey design and participants

The survey was developed based on a review of relevant literature and consultation with experienced MSL leaders and Medical Affairs professionals.

The survey consisted of 14 questions organized across key domains, including AI awareness, current utilization, perceived benefits and challenges, and organizational policies related to AI use in Medical Affairs. The instrument was developed based on prior MSL Society surveys and subject matter expertise and underwent internal review by experienced MSL leaders and Medical Affairs professionals to assess clarity and relevance. Pilot testing with a small group of Medical Affairs leaders resulted in minor refinements to wording and structure to improve clarity, without substantive changes to the overall survey design.

The questionnaire consisted of closed-ended categorical-response items, including nominal and ordinal multiple-choice questions, designed to assess organizational practices for deploying MSL teams. The finalized survey was administered online over a three-week period.

This study utilized a cross-sectional survey design. The survey targeted individuals in Medical Affairs, including MSLs, MSL managers and directors, MSL excellence and operations, executive leadership, and other roles.

The survey was designed to address several key research questions. Specifically, it aimed to assess the current level of AI adoption and utilization among MSLs globally, as well as to explore how Medical Affairs professionals perceive the benefits and potential of integrating AI into their roles. It also examined the extent to which pharmaceutical companies are establishing governance frameworks for the implementation of AI within Medical Affairs. Finally, the survey considered the anticipated future impact of AI on MSL activities, including interactions with KOLs.

Inclusion criteria

Although the survey was open to a broad audience, this analysis only included participants who identified as currently being in one of the following Medical Affairs roles: executive management/vice president of Medical Affairs, manager/director of MSLs (or equivalent title), MSL operations/excellence/training, or MSL/senior MSL/medical advisor (or equivalent title). Responses from individuals outside these roles were excluded to ensure that the analysis reflected professionals with direct, relevant experience in Medical Affairs. The respondents self-selected to participate, and all responses were analyzed anonymously.

Survey development and distribution

The MSL Society developed and administered the survey. Prior to distribution, MSL Society leadership and subject matter experts reviewed the survey instrument to assess content validity, clarity, and relevance to Medical Affairs operations. The review included experienced MSL leaders and Medical Affairs professionals, including individuals in leadership roles such as directors and vice presidents, representing diverse perspectives within the function.

The survey used a non-probability convenience sampling approach targeting experienced Medical Affairs and MSL professionals. The participants were recruited through LinkedIn, MSL Society newsletter subscribers, pharmaceutical industry conferences, and online platforms dedicated to Medical Affairs professionals. Due to the nature of distribution through open professional networks and electronic communications, the total number of individuals who received or viewed the survey invitation could not be determined; therefore, a formal response rate was not calculated. Only submitted surveys were included in the final dataset; therefore, information on incomplete responses or survey dropouts was unavailable. Because the survey relied on self-selection and convenience sampling, the results may be subject to potential response bias, including self-selection bias among individuals with greater interest or experience in Medical Affairs and AI. This study utilized non-probability convenience sampling with voluntary participation, which may introduce selection bias and limit the generalizability of the findings to the broader global Medical Affairs population. As such, the results should be interpreted as descriptive and exploratory, reflecting the respondents' perspectives rather than a fully representative sample.

Data analysis

Data were collected using the GoTo® survey software (GoTo Group, Inc., Boston, MA). Survey responses were compiled and analyzed using descriptive statistical methods. Data analysis was conducted using Microsoft Excel (Microsoft Corporation, Redmond, WA).

For each survey question, results are presented as the percentage of respondents selecting each response option. All survey questions were required; therefore, the number of respondents was consistent across all items, and results are presented using descriptive statistics, including frequencies and percentages. Because the study was designed as a descriptive survey analysis, no formal sample size calculation was performed. The objective of the analysis was to summarize the respondents' characteristics, organizational practices, and perceptions regarding the launch of MSL teams, rather than to test statistical hypotheses. Inferential statistical testing and subgroup analyses were not performed because the study did not aim to test predefined hypotheses, compare predefined subgroups, or establish causal relationships, and responses were intentionally analyzed in aggregate rather than stratified by role, geography, or company type.

The study design and reporting followed established best practices for online survey research and were consistent with the Checklist for Reporting Results of Internet E-Surveys (CHERRIES) guidelines [15]. Data quality checks were performed prior to analysis, including screening for duplicate responses. Only fully completed responses were included in the analysis.

Ethics approval and consent to participate

This study involved an anonymous, voluntary survey of global Medical Affairs professionals. No patient data, personally identifiable information (PII), or sensitive health information was collected. Based on the nature of the study and applicable guidelines for survey-based research, formal institutional review board (IRB) or ethics committee approval was not required. This determination is consistent with prevailing international guidelines, including the Declaration of Helsinki, the Council for International Organizations of Medical Sciences (CIOMS) guidelines, and the US Common Rule. Participation was voluntary, and informed consent was implied through the completion of the questionnaire.

## Results

A total of 584 responses were collected from 58 countries. After applying the inclusion criteria, 367 responses from 48 countries were included in the final analysis. The results are presented in two parts: the characteristics of the survey respondents (including geographic distribution, roles, company type, and years of experience), followed by findings on AI utilization, including whether the participants currently use AI tools, the frequency of use, primary applications, perceived benefits, organizational policies and broader adoption, personal awareness, perceived threats to the MSL role, anticipated future impact, and the importance of learning AI to remain competitive. All the results are reported as frequencies and percentages.

The participants represented diverse countries and regions, with the largest proportion from the United States (63%), followed by Canada (8%), the United Kingdom (3%), and Germany, Pakistan, and Spain (2% each). Many other countries each accounted for less than 1% of responses (Table 1).

**Table 1:** Country/region

Category	Percentage
Armenia	<1%
Australia	<1%
Austria	<1%
Bangladesh	<1%
Belgium	1%
Brazil	1%
Bulgaria	<1%
Cameroon	1%
Canada	8%
Costa Rica	<1%
Cote d'Ivoire	<1%
Dominican Republic	<1%
Ecuador	<1%
Egypt	1%
France	1%
Germany	2%
Greece	1%
India	1%
Iran	<1%
Ireland	<1%
Italy	1%
Japan	<1%
Jordan	<1%
Kazakhstan	1%
Kuwait	<1%
Libya	<1%
Malaysia	<1%
Mexico	1%
Myanmar	<1%
Nigeria	1%
Norway	<1%
Pakistan	2%
Philippines	<1%
Poland	1%
Portugal	1%
Puerto Rico	1%
Saudi Arabia	<1%
South Africa	<1%
South Korea	1%
Spain	2%
Switzerland	1%
Syria	<1%
Taiwan	1%
Turkey	1%
Ukraine	<1%
United Arab Emirates	1%
United Kingdom	3%
United States	63%

The majority of the respondents (62%) reported their current role as an MSL, senior MSL, or medical advisor. Managers or directors of MSLs accounted for 18%, MSL operations/excellence/training for 11%, and executive management or vice president of Medical Affairs for 9% (Table 2).

**Table 2:** What is your current role? MSLs: medical science liaisons

Category	Percentage
Executive management/vice president of Medical Affairs	9%
Manager/director of MSLs (or equivalent title)	18%
MSL operations/excellence/training	11%
MSL/senior MSL/medical advisor (or equivalent title)	62%

The largest proportion of respondents (34%) reported working in a large pharmaceutical/biotechnology company, followed by 22% in a small pharmaceutical company and 21% in a medium-sized organization. Medical device and other companies each represented 7%, diagnostic companies 5%, contract research organizations 3%, and contract MSL organizations 2% (Table 3).

**Table 3:** How would you classify your company? MSL: medical science liaison

Category	Percentage
Large pharmaceutical/biotechnology (revenue of 10+ billion USD)	34%
Small pharmaceutical/biotechnology (revenue less than 1 billion USD)	22%
Medium pharmaceutical/biotechnology (revenue of 1-10 billion USD)	21%
Medical device	7%
Others	7%
Diagnostic company	5%
Contract research organization (CRO)	3%
Contract MSL organization	2%

Twenty-four percent of the respondents reported 2-4 years of experience in their current function, followed by 17% with 1-2 years, 14% with 4-6 years, and 12% with less than one year. Eleven percent reported more than 15 years of experience, 9% had 6-8 years, 8% had 10-15 years, and 5% reported 8-10 years of experience (Table 4).

**Table 4:** How many total years of experience do you have in your current function (i.e., as an MSL/medical advisor, MSL operations, or MSL leadership)? Include all previous companies where you worked only in the same function, if applicable MSL: medical science liaison

Category	Percentage
Less than one year	12%
1-2 years	17%
2-4 years	24%
4-6 years	14%
6-8 years	9%
8-10 years	5%
10-15 years	8%
More than 15 years	11%

AI adoption for KOL engagement remains low, with the majority of the respondents either not using these tools (60%) or reporting no knowledge of them (13%) (Table 5).

**Table 5:** Do you, or the MSLs that you manage, currently utilize AI tools in preparation for KOL engagements? MSLs, medical science liaisons; AI, artificial intelligence; KOL, key opinion leader

Category	Percentage
Yes	27%
No	60%
I have no knowledge of this	13%

The survey revealed that 42% of MSLs, senior MSLs, and medical advisors never use AI tools to prepare for KOL engagements, 27% report rarely using them, and 20% report occasional use. Another 10% reported frequent use, and 1% reported always using AI tools (Table 6).

**Table 6:** How often do you, or the MSLs that you manage, utilize AI tools to prepare for KOL engagements? MSLs, medical science liaisons; AI, artificial intelligence; KOL, key opinion leader

Category	Percentage
Always	1%
Frequently	10%
Occasionally	20%
Rarely	27%
Never	42%

The respondents primarily leverage AI for literature reviews, data analysis, and presentation preparation (58%). Fourteen percent reported using AI tools for real-time information retrieval during KOL engagements, and 10% selected others. Nineteen percent indicated that AI tools were not being used (Table 7).

**Table 7:** Which task do you or would you, or the MSLs you manage, primarily leverage AI tools for? Select one MSLs, medical science liaisons; AI, artificial intelligence; KOL, key opinion leader

Category	Percentage
Literature review	22%
Data analysis	20%
AI tools are not utilized	19%
Presentation preparation	16%
Real-time information retrieval during KOL engagements	14%
Others	10%

Improved efficiency is cited as the primary benefit of AI usage (42%), significantly outperforming other advantages such as enhanced KOL preparation and data analysis. Despite these perceived gains, a notable portion of the cohort continues to report no AI utilization (Table 8).

**Table 8:** What is the primary benefit you, or the MSLs you manage, have gained or could gain from using AI tools? Select one MSLs, medical science liaisons; AI, artificial intelligence; KOL, key opinion leader

Category	Percentage
Improved efficiency	42%
AI tools are not utilized	21%
Better preparation for KOL engagements	18%
Enhanced data analysis	13%
Increased accuracy in information delivery	4%
Others	3%

Comprehensive AI policies are rare among represented organizations. Most respondents reported either having some policies in place, being in the process of developing them, or having no formal policy (Table 9).

**Table 9:** Does your organization currently have any policies or procedures governing the use of AI by MSLs? MSLs, medical science liaisons; AI, artificial intelligence

Category	Percentage
Yes, comprehensive policies	8%
Yes, some policies	25%
No, but they are being developed	9%
No, there are no policies	27%
I am not sure	31%

Nearly half of the respondents (47%) report that their organizations utilize AI. An additional 14% indicated no utilization, while 39% were unsure (Table 10).

**Table 10:** To your knowledge, are any AI tools being utilized (or soon to be) within your broader organization (within Medical Affairs and/or other functions)? AI: artificial intelligence

Category	Percentage
Yes	47%
No	14%
I am not sure	39%

The survey also revealed that only 6% of the respondents are very knowledgeable about AI, 14% were knowledgeable, 41% were somewhat knowledgeable, 30% were not very knowledgeable, and 9% were not at all knowledgeable (Table 11).

**Table 11:** How would you rate your own personal awareness of the concept of AI and/or any AI tools? AI: artificial intelligence

Category	Percentage
Very knowledgeable	6%
Knowledgeable	14%
Somewhat knowledgeable	41%
Not very knowledgeable	30%
Not at all knowledgeable	9%

While most respondents do not view AI as a threat to MSLs' core activities (58%), a substantial minority is unsure (Table 12).

**Table 12:** Do you perceive AI as a threat to any of the typical activities of the MSL role? MSLs, medical science liaisons; AI, artificial intelligence

Category	Percentage
Yes	11%
No	58%
I am not sure	31%

Most respondents expect AI to have an impact in the future, with 74% reporting that it would be highly impactful or impactful, while 5% reported limited or no impact (Table 13).

**Table 13:** How would you assess the potential future impact of AI in Medical Affairs and the MSL function? MSLs, medical science liaisons; AI, artificial intelligence

Category	Percentage
Highly impactful	27%
Impactful	47%
Somewhat impactful	21%
Limited impact	4%
No impact	1%

The survey revealed that 57% of MSLs, senior MSLs, and medical advisors indicated that learning about AI technology was very important for remaining competitive. In contrast, 1% indicated that it was not very important, and fewer than 1% reported that it was not at all important (Table 14).

**Table 14:** In general, how important is it to learn about how the latest AI technology can assist MSLs and Medical Affairs to stay competitive and keep up with the rapidly changing landscape? MSLs, medical science liaisons; AI, artificial intelligence

Category	Percentage
Very important	57%
Important	30%
Somewhat important	12%
Not very important	1%
Not at all important	<1%

## Discussion

The survey results provide a comprehensive overview of AI adoption within Medical Affairs, with particular emphasis on MSLs and their interactions with KOLs. The findings reveal a nuanced landscape with significant implications for the future of the MSL profession, as well as other functions within Medical Affairs. While direct comparisons are limited given the absence of prior MSL-specific studies, surveys of AI adoption in broader healthcare contexts, such as clinical practices, diagnostics, and administrative processes, have similarly reported early-stage adoption patterns, with enthusiasm tempered by barriers, including governance limitations, the readiness of the workforce, and infrastructure constraints [9,10,12,13,15-19]. Notably, studies have identified challenges spanning external system-level conditions, strategic change management capacity, and shifts in professional roles and workflows [20]. Other research among clinicians emphasizes similar barriers: limited AI literacy, transparency concerns, and anxiety over role displacement, as well as the importance of organizational support and leadership in facilitating the adoption of AI technologies in clinical practice [12,13,21]. These parallels suggest that MSLs confront challenges consistent with broader trends in healthcare AI implementations.

These findings provide important context within the broader trends of AI adoption in healthcare and the pharmaceutical industry, where increasing interest in AI is often accompanied by variability in implementation, governance, and perceived value [18-22]. The relatively low reported use of AI among MSLs, despite recognized benefits such as improved efficiency, suggests that barriers, including a lack of organizational policies, limited training, and uncertainty about appropriate use, may be contributing to slower adoption. From a practical perspective, these results highlight the need for Medical Affairs to establish clear guidance, provide targeted training, and define appropriate use cases for AI to support MSL activities. In addition, the findings reinforce the importance of maintaining the human element in scientific exchange, ensuring that AI serves as a supportive tool rather than a replacement for scientific expertise and critical thinking.

These challenges may also be impacting MSL adoption, as our survey found that only 27% of MSLs, senior MSLs, and medical advisors currently utilize AI tools in preparation for KOL engagements, while the majority (60%) reported no use. This disparity suggests a potential "innovation gap" within the MSL profession, where early adopters may gain competitive advantages while others risk falling behind in leveraging AI's benefits.

The applications of AI for MSLs, senior MSLs, and medical advisors span multiple aspects of their role, with the technology primarily leveraged for literature review (22%), data analysis (20%), and the preparation of presentations (16%). These tasks represent core responsibilities in KOL scientific exchange and engagement, suggesting that AI is primarily being used to support information gathering, analysis, and communication rather than replace the interpersonal elements of the role. While adoption remains relatively low, the findings demonstrate the diverse potential of AI to enhance MSL activities and highlight the substantial opportunity for broader integration in the future.

The perceived benefits of AI for MSLs, senior MSLs, and medical advisors align with broader digital transformation trends in healthcare [8,10,12,14-16,18,20,21]. The most commonly cited advantages were improved efficiency (42%) and better preparation for KOL engagements (18%). These benefits could help address persistent challenges in the profession, such as information overload and the need for rapid, data-driven decision-making [22-24]. Although efficiency is important and the use of AI can enhance the effectiveness of MSLs, it cannot replace the interpersonal competencies of emotional intelligence and nuanced communication required to build trust and maintain value-added relationships with KOLs [25-27].

The survey also revealed substantial gaps in AI implementation and governance for MSLs. Limited adoption, coupled with uncertainty regarding organizational policies (with 31% of the respondents reporting that they were unsure), highlights the need for more clearly defined strategies. Notably, only 33% of the respondents reported that their companies have established policies governing AI use by MSLs, reflecting a governance gap that may contribute to inconsistent practices and increase the risk of inappropriate application of AI tools.

The respondents' knowledge of AI varied significantly, with 20% describing themselves as very knowledgeable or knowledgeable and 39% as not very or not at all knowledgeable. This disparity highlights the need for comprehensive training focused on the effective application and appropriate utilization of AI in the MSL role.

Despite these challenges, there is an awareness of AI's potential impact on the MSL role. In fact, the majority (87%) of the respondents considered it important or very important to learn about AI to remain competitive in their role. This recognition suggests that MSLs are cognizant of the transformative potential of AI. As the technology becomes more sophisticated and more deeply integrated into Medical Affairs, the MSL role is likely to evolve; however, human judgment and the ability to foster valuable and strategic relationships with KOLs will remain indispensable. Collectively, these findings highlight both the potential and complexity of AI adoption in the MSL profession and, more broadly, across Medical Affairs.

Despite the potential benefits of AI, several important risks must be considered in the context of Medical Affairs. AI-generated scientific content may contain inaccuracies, incomplete interpretations, or fabricated information, which could impact the quality of scientific exchange if not carefully verified. In addition, the use of AI in interactions with KOLs and HCPs raises important compliance and regulatory considerations, particularly regarding the use of approved content, data privacy, and alignment with organizational policies. There is also a risk of overreliance on AI tools in high-stakes scientific discussions, which may reduce critical thinking and scientific rigor if not balanced with appropriate human expertise. These findings highlight the importance of establishing clear guidelines, training, and governance to ensure the responsible and appropriate use of AI within Medical Affairs.

Limitations

This study has several limitations that should be considered when interpreting the findings. First, the results are based on self-reported data, which may be subject to recall bias or social desirability bias. Although anonymity was assured to encourage candid responses, the reported perceptions may not fully reflect actual practices. Second, while the survey included respondents from 48 countries and regions, the geographic distribution was uneven, with certain regions being more heavily represented than others. This imbalance may limit the generalizability of the findings to underrepresented areas. Third, the cross-sectional design provides a snapshot of perceptions at a single point in time. Given the rapid pace of AI development, adoption patterns and attitudes may have evolved since the survey was conducted. Finally, the use of a non-probability convenience sample with voluntary participation may affect the generalizability of these findings to the broader global Medical Affairs population. As such, the results should be interpreted as descriptive and exploratory, reflecting the respondents' perspectives rather than a fully representative sample.

Regarding data analysis, this study relied primarily on descriptive statistics, which provide valuable insights into patterns of adoption and perceptions but do not allow for hypothesis testing or the identification of causal relationships. Although appropriate for this exploratory research, the lack of inferential analysis restricts the ability to draw conclusions about associations between participant characteristics and AI adoption. In addition, as participation was voluntary, the sample may be subject to response bias, with individuals more interested in AI or digital innovation being more likely to complete the survey. This potential bias should be considered when interpreting the results, as it may overrepresent enthusiasm for AI adoption.

Additionally, because the survey was distributed through open professional channels, the total number of individuals who received the survey invitation could not be determined, and a response rate could not be calculated, which may limit the assessment of representativeness and introduce potential nonresponse bias. Future research may build upon these findings by conducting subgroup analyses or applying inferential methods to examine differences between respondent categories. Despite these limitations, this study is the first to examine AI adoption and perceptions among MSLs and other roles in Medical Affairs.

Future research

Future studies should build on these findings to provide deeper insights. Longitudinal research could track changes in AI adoption and perceptions over time, clarifying long-term trends in Medical Affairs. Comparative studies across different regions, company sizes, or therapeutic areas could uncover contextual factors that influence adoption and implementation. Additional research on specific AI applications, such as predictive analytics, natural language processing for literature review, and real-time information support during KOL interactions, would help to evaluate their effectiveness and practical value.

Further investigations into organizational readiness and governance frameworks are warranted. The survey revealed substantial gaps in awareness and policy, underscoring the need for best practices that address transparency, compliance, and data privacy. In parallel, research should focus on the evolving competencies required of MSLs in an AI-enabled environment, helping to inform structured training programs and professional development strategies. Finally, the ethical implications of AI adoption, including potential bias in algorithms, data ownership, and the effect on human-centered scientific exchange, deserve closer attention. Such research is critical to ensure that AI integration strengthens rather than diminishes the human-centered role of MSLs in advancing scientific exchange and contributing to improved patient outcomes.

## Conclusions

This global survey provides the first comprehensive examination of the adoption and perceptions of AI within Medical Affairs, with a particular focus on medical science liaisons. Adoption remains limited, with only 27% of MSLs, senior MSLs, and medical advisors currently using AI tools when preparing for a KOL engagement, with improved efficiency and better preparation for KOL interactions emerging as the most valued benefits. However, significant gaps persist in organizational governance, policy frameworks, and AI-related training. Notably, only a minority of organizations have established policies governing AI use by MSLs, reflecting variability in implementation and oversight. Despite these gaps, nearly three-quarters of respondents believe AI will be impactful or highly impactful for the future of Medical Affairs and the MSL function. In addition, the vast majority of respondents consider it important or very important to learn about AI technology to remain competitive. Together, these findings highlight both an urgent need and a timely opportunity for organizations to establish structured governance, invest in targeted training, and explore practical implementation strategies. As AI technologies continue to evolve, their integration should be guided by responsible frameworks that ensure appropriate use, compliance, and scientific rigor. Importantly, the human-centered aspects of the MSL role, including scientific exchange, trust, and relationship building with KOLs, remain essential and cannot be replaced. The relational and communication-based aspects of the MSL role, grounded in trust, dialogue, and emotional intelligence, will continue to be indispensable, reinforcing the importance of human judgment in fostering valuable and strategic relationships with KOLs.

## Appendices

Supplementary material 1: Survey

1.     Country/region

2.     What is your current role?

3.     How would you classify your company?

4.     How many total years of experience do you have in your current function (i.e., as an MSL/medical advisor, MSL operations, or MSL leadership)?

5.     Do you, or the MSLs that you manage, currently utilize AI tools in preparation for KOL engagements?

6.     How often do you, or the MSLs that you manage, utilize AI tools to prepare for KOL engagements?

7.     Which task do you or would you, or the MSLs you manage, primarily leverage AI tools for?

8.     What is the primary benefit you, or the MSLs you manage, have gained or could gain from using AI tools?

9.     Does your organization currently have any policies or procedures governing the use of AI by MSLs?

10.   To your knowledge, are any AI tools being utilized (or soon to be) within your broader organization (within Medical Affairs and/or other functions)?

11.    How would you rate your own personal awareness of the concept of AI and/or any AI tools?

12.   Do you perceive AI as a threat to any of the typical activities of the MSL role?

13.   How would you assess the potential future impact of AI in Medical Affairs and the MSL function?

14.   In general, how important is it to learn about how the latest AI technology can assist MSLs and Medical Affairs to stay competitive and keep up with the rapidly changing landscape?
